# B2 microglobulin is a novel prognostic marker of Angioimmunoblastic T-cell lymphoma

**DOI:** 10.1038/s41598-018-31212-z

**Published:** 2018-08-27

**Authors:** Yufeng Shang, Xiaorui Fu, Yu Chang, Yanan Li, Mingzhi Zhang

**Affiliations:** 1 Department of Oncology, the First Affiliated Hospital of Zhengzhou University, Zhengzhou, Henan China; 2 Lymphoma Diagnosis and Treatment Center, Zhengzhou, Henan China; 3 Institute of Translational Medicine, Zhejiang University, Hangzhou, Zhejiang China

## Abstract

The aim of the present study was to analyze features and explore parameters that can help to predict prognosis for angioimmunoblastic T-cell lymphoma (AITL). A total of 117 patients with AITL were retrospectively analyzed. Multivariate analysis showed that β2 microglobulin (β2-M) ≥4.0 mg/L (*P* = 0.020), rash/pruritus (*P* = 0.004), performance status (PS) ≥2 (*P* = 0.006), age >60 years (*P* = 0.006) and extranodal sites (ENSs) >1 (*P* = 0.029) were independent risk factors for OS. Rash/pruritus (*P* = 0.007), age >60 years (*P* = 0.035) and ENSs >1 (*P* = 0.006) were independent risk factors for PFS. A novel prognostic model consisting of β2-M, rash/pruritus, PS, age and ENSs >1 was constructed. The model classified patients into 3 risk stratifications: low risk (0 or 1 factor), intermediate risk (2 factors), high risk (≥3 factors) and significantly stratified patients with AITL (*P* < 0.001). In conclusion, except for PS ≥2, age >60 years and ENSs >1 used in IPI, β2-M and rash/pruritus also indicated adverse prognosis. That we constructed model was commendably prognostic for OS and PFS.

## Introduction

Angioimmunoblastic T-cell lymphoma (AITL) derives from T follicular helper cell expressing CXCL13, CD10 and PD-1 and has unique clinical and pathological features^1,2^. AITL mainly affected aged individuals with the median age of 60 years^3^. It usually accompanies Epstein-Barr virus (EBV) infection and dysregulation of immune system, distinct rash or pruritus onset. Hypoalbuminemia, hypergammaglobulinemia, anemia, lymphocytopenia, eosinophilia and positive Coombs test commonly appear, representing characteristic manifestations of AITL^4^.

The disease generally displays aggressive clinical course and poor prognosis with 5-year OS rate less than 40% in spite of tackled with combined chemotherapy^4,5^. Unlike other subtypes of lymphoma such as diffuse large B cell lymphoma (DLBCL) and follicular lymphoma which have their own prognostic model, prognostic factors or predictive model for AITL have not been constructed. International Prognostic Index (IPI), which is applied to DLBCL in previous studies, is controversial for the administration to T-cell lymphoma^6^. Although the prognostic model for peripheral T-cell lymphoma, not otherwise specified (PIT) has been proposed, it did not appear to be clinically more useful than the original IPI^7,8^. Some studies have attempted to explore the prognostic risk factors and new prognostic model for AITL, but yielding controversial results^3,4,9,10^. In this study, 117 patients with AITL were retrospectively analyzed to summary characteristics and explore prognostic factors.

Beta2 microglobulin (β2-M) is an invariant subunit of the class I human leukocyte antigen complex (HLA-I) on the plasma membrane of most nucleated cells. The best characterized function of β2-M is to interact with and stabilize the tertiary structure of the MHC class I a chain and involve the presentation of antigenic peptides which were then recognized by the T-cell receptors of CD8+ cytotoxic T lymphocytes (CTL)^11,12^. Study has confirmed that β2-M played a significant role in evaluating prognosis of non-Hodgkin lymphoma^13^. B2-M has also been incorporated into novel or modified prognostic model for more accurate prognostication to identify different risk groups in follicular lymphoma and diffuse large B-cell lymphoma^14,15^. However, its role in predicting clinical outcomes in AITL has not been extensively investigated. In this retrospective study, we evaluated the prognostic value of B2-M for patients with AITL.

## Methods

### Patients

In all, 117 patients newly diagnosed with AITL and treated at the first affiliated hospital of Zhengzhou University were enrolled from April 2011 to June 2016 in this retrospective study. All patients had a pathological diagnosis of AITL according to the World Health Organization lymphoma classification criteria, as determined by two or more expert hematopathologists. *In situ* hybridization for EBV-encoded early small RNA (EBER) were also performed on these specimens. These patients had complete medical history and clinical dates, including laboratory examination, computed tomographic (CT) scan imaging of chest and abdomen and pelvis or positron emission tomography-computed tomography (PET-CT) of the whole body to perfect Ann Arbor stage. Laboratory examination contained peripheral blood cell count, liver and kidney function, lactate dehydrogenase (LDH) and β2-M. Cases were scored by IPI scores (scoring system including age >60 years, PS ≥2, LDH over normal, Ann Arbor stage III or IV, ENSs >1) and PIT scores (scoring system including age >60 years, PS ≥2, LDH over normal, and bone marrow involvement)^6,7^. Performance status(PS) was evaluated based on the Eastern Cooperative Oncology Group scale^16^. Age, B symptom and rash/pruritus also were recorded.

The study was approved by Ethics Committee for Scientific Research and Clinical Trials of Zhengzhou University and informed consent was obtained from the patients.

### Treatment Response Evaluation

Treatment responses including complete remission (CR), partial remission (PR), stable disease (SD), and progressive disease (PD) were evaluated according to the response criteria of Cheson *et al*.^17^. The evaluation to prognostic included overall survival (OS) and progression-free survival (PFS). OS was defined as the time from diagnosis to last follow-up or death resulting from any cause. PFS was calculated from the date of diagnosis to the date of disease progression, relapse and death from any cause or the last follow-up. Follow-up of patients not experiencing any of these events was censored at the date of last contact.

### Statistical Analysis

Statistical analyses were performed using IBM SPSS statistics software, version 21.0 and GraphPad Prism 6. OS and PFS distributions were estimated using the Kaplan–Meier curve analysis, time-to-event distributions were compared using the log-rank test and two-tailed significance-level of 0.05 was considered statistically significant. Comparisons of clinical and prognostic factors were performed using the Pearson’s chi-squared test. Multivariate analysis was performed with a Cox hazards regression model using forward/backward stepwise method with the use of threshold values for removal from and addition to the model of *P* = 0.10 and *P* = 0.05 respectively. Results are expressed as hazard ratios (HRs) and 95% confidence intervals (CIs).

## Results

### Patient Characteristics

We analyzed 117 patients and concluded general characteristics in the Table 1. The median age was 62 years old ranging from 19 to 84 years with a male-to-female ratio of 1.85:1. In all, 110 patients encountered advanced-stage III-IV and ENSså 1 took up 46.0% (52/113). PS ≥2 was in 16.2% (19/117). B symptoms and rash/pruritus were observed in 66.7% (78/117) and 29.9% (35/117) respectively. The patients who suffered rash/pruritus frequently accompanied B symptoms (29/35) and there was a significant correlation between the two symptoms (*P* = 0.015). The serum LDH levels elevated (>245 U/L) in 75.0% patients and the serum β2-M levels elevated (>4 mg/L) in 58.3% patients. The IPI scores in 70.8% (80/113) patients were more than 2 and the PIT scores in 56.0% (56/100) patients were more than 2.

**Table 1:** Clinical characteristics of 117 patients with AITL.

Characteristic	Median	Rang	No.	%
Male sex			76/117	65.0
Age (year)	62	19–84		
Age >60years			67/117	57.3
ECOG PS ≥2			19/117	16.2
Stages III or IV			110/113	97.3
Stage IV			65/113	57.5
Extranodal involvement			80/113	70.8
Extranodal sites >1			52/113	46.0
BM involvement			16/100	16.0
Liver involvement			15/113	13.3
Spleen involvement			51/113	45.1
B symptoms			78/117	66.7
Rash/Pruritus			35/117	29.9
Hemoglobin (g/L)	109	39–160		
Anemia*			86/117	73.5
Platelet (x10^9^/L)	172	11–466		
<150 × 10^9^/L			43/117	36.8
Lymphocyte (x10^9^/L)	1.0	0.2–6.9		
≤1.1 × 10^9^/L			65/117	55.6
Eosinophil (x10^9^/L)	0.16	0–2.34		
≥0.5 × 10^9^/L			22/117	18.8
Monocyte (x109/L)	0.62	0.02–2.4		
≥0.6 × 109/L			59/117	50.4
LDH (U/L)	301	126–1020		
LDH level ≥245 U/L			87/116	75.0
β2 microglobulin (mg/L)	4.38	1.22–14.87		
β2 microglobulin level ≥4.0 mg/L			67/115	58.3
Serum albumin (g/L)	33.55	16.2–46.3		
Serum albumin level ≤35 g/L			64/114	56.1
Serum globulin (g/L)	30.9	17.3–70		
Serum globulin level ≥35 g/L			42/114	36.8
**IPI score**				
Low risk			7/113	6.2
Low-intermediate risk			26/113	23.0
High-intermediate risk			44/113	38.9
High risk			36/113	31.9
**PIT score**				
Group 1			8/100 8.0	
Group 2			36/100 36.0	
Group 3			42/100 42.0	
Group 4			14/100 14.0	
**Pathological finding**				
EBER (+)			49/69	71.0
CXCL13 (+)			57/77	74.0
BCL-6 (+)			66/78	84.6
CD10 (+)			61/92	66.3
PD-1 (+)			28/29	96.6

Abbreviations: BM, bone marrow; EBER, EBV-encoded early small RNA;ECOG PS, Eastern Cooperative Oncology Group performance status; IPI, International Prognostic Index; LDH, lactic dehydrogenase; PIT, Prognostic Index for Peripheral T-Cell Lymphoma, Unspecified.

*The presence of anemia was defined as the value of hemoglobin level <130 g/L for men and 115 g/L for women because the lower limit of normal was 130 g/L for men and 115 g/L for women based on our examination.

Total 69 cases were performed EBER *in situ* hybridization. Twenty cases (29.0%) were EBV negative and the other 49 cases (71.0%) contained a variable number of EBER-positive cells. The results of immunohistochemical staining showed that CXCL 13 (+), CD10 (+), BCL6 (+) and PD1 (+) were in 74.0% (57/77), 66.3% (61/92), 84.6% (66/78) and 96.6% (28/29) patients respectively.

### Treatment and Survival

In 117 patients, 17 patients lost to follow-up after diagnosis without treatment. Total 64 patients received the anthracycline containing arms as the first-line treatment. Among the 64 patients, 58 patients received CHOP regimen, 2 patients received CHOPE regimen, 2 patients received EPOCH regimen, 1 patients received Hyper CVAD regimen, 1 patients received M-BOCAD (Methotrexate, Bleomycin, Cyclophosphamide, Vincristine and Pirarubicin) regimen. Total 30 patients accepted combination chemotherapy without an anthracycline but based on Gemcitabine and Platinum arms which plus prednisone or thalidomide. Three patients received oral chemotherapy containing Cyclophosphamide and Thalidomide and another 3 patients did never get any therapy and were observed only. Primary treatment failure changed to another therapy including chemotherapy and/or autologous or allogeneic treatment.

**Figure 1:**
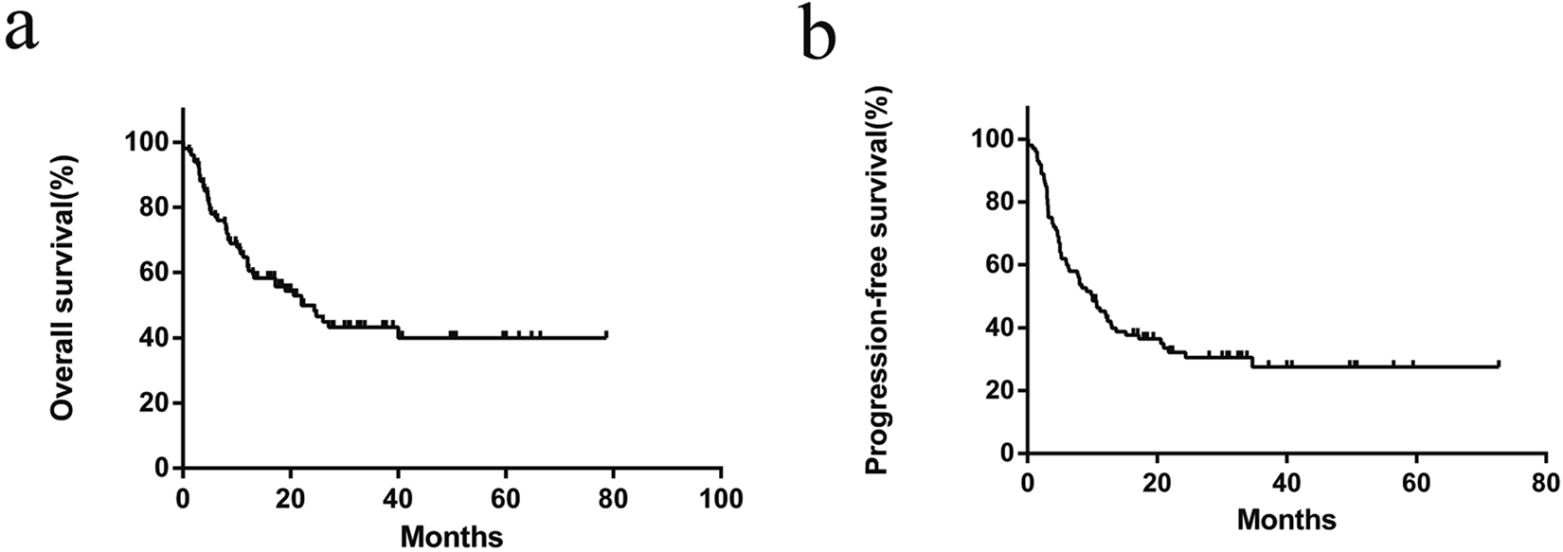
Overall survival (**a**) and progression-free survival (**b**) of 100 patients followed up among 117 patients with angioimmunoblastic T-cell lymphoma.

In the eligible 100 patients followed up among 117 patients, the median survival time was 22 months. 3-year OS rate for the entire group was 43.3%, and 3-year PFS rate was 27.5% (Fig. 1). Of the 97 cases with treatment, the ORR (overall response rate) was 77.3%, with CR and PR rates of 26.8% and 50.5%. In cases treated with anthracycline containing arms, 3-year OS and PFS rate was 45.1% and 26.4% and with that of 47.5% and 36.5% for gemcitabine and platinum based arms. But there was no statistical significance (*P* = 0.816) in the two groups. For the patients with oral chemotherapy or without treatment, the longest survival time was 3.2 months.

### Univariate and Multivariate Analysis for Prognostic Factors

We performed univariate analysis to evaluate the prognostic factors for AITL. The following factors predicted poor OS: rash/pruritus (*P* = 0.001), PS ≥2 (*P* = 0.000), liver involvement (*P* = 0.035), platelet count <150 × 10^9^/L (*P* = 0.045) and β2-M ≥4.0 mg/L (*P* = 0.000). Rash/pruritus (*P* = 0.002), β2-M >4.0 mg/L (*P* = 0.010), ENSs >1 (*P* = 0.007), Monocytoza (*P* = 0.021), PS ≥2 (*P* = 0.042) and serum globulin level over 35 g/L (*P* = 0.037) leaded to an inferior PFS (Table 2). We also analyzed the link between survival and EBV, the expression of CXCL13, BCL-6, CD10 and PD-1, there were no statistical significance respectively (Table 2). Although age, LDH level and Ann Arbor stage had no statistical significance to OS and PFS in univariate analysis, they were still inclusive into multivariate analysis on account of their clinical significance. Cox multivariate analysis concluded that β2-M ≥4.0 mg/L (*P* = 0.020), rash/pruritus (*P* = 0.004), age >60(P = 0.006), PS ≥2 (*P* = 0.006) and ENSs >1 (*P* = 0.029) were the independent risk factors for OS, rash/pruritus (*P* = 0.007), age >60(*P* = 0.035) and ENSs >1 (*P* = 0.006) were the independent risk factors for PFS (Table 3).

**Table 2:** Univariate analysis according to 100 patients’ characteristics.

Characteristic	No.	3-year OS %	P(Univariate Analysis to OS)	3-year PFS %	P(Univariate Analysis to PFS)
**Gender**					
Male	66	39.9	0.331	27.6	0.577
Female	34	50.3		28.9	
**Age**					
≤60 years	42	55.9	0.053	42.8	0.072
> 60 years	58	33.1		15.6	
**PS**					
0–1	79	48.6	0.000	27.6	0.042
2–4	21	22.9		23.8	
**Ann Arbor stage**					
I–II	2	50.0	0.774	50.0	0.807
III–IV	98	43.1		28.1	
**No. of extranodal sites**					
0–1	56	55.3	0.080	38.0	0.007
> 1	44	30.3		14.8	
**BM involvement**					
Yes	14	45.9	0.063	26.8	0.313
No	72	31.3		29.3	
**Liver involvement**					
Yes	11	13.6	0.035	18.2	0.360
No	89	47.2		29.1	
**Spleen involvement**					
Yes	43	39.1	0.539	27.3	0.928
No	57	46.6		22.0	
**B symptom**					
Yes	64	36.8	0.166	31.2	0.916
No	36	58.3		18.1	
**Rash/Pruritus**					
Yes	29	25.4	0.001	18.7	0.002
No	71	50.8		31.5	
Anemia*					
Yes	69	38.8	0.372	26.6	0.764
No	31	53.3		30.7	
**Platelet count**					
≥150 × 10^9^/L	65	48.0	0.045	31.3	0.239
<150 × 10^9^/L	35	33.8		22.8	
**Lymphocyte**					
normal	42	48.6	0.710	28.3	0.457
≤normal	58	40.2		32.5	
**Eosinophil**					
≥normal	19	39.0	0.648	26.3	0.481
normal	81	44.3		27.6	
**Monocyte**					
≥normal	53	34.2	0.219	18.4	0.021
normal	47	53.5		37.4	
**LDH level**					
≥normal	75	36.9	0.166	28.7	0.223
normal	25	58.4		30.6	
**β2-M level**					
≥4.0 m g/L	56	26.2	0.000	21.6	0.010
<4.0 m g/L	44	65.7		35.9	
**Serum albumin level**					
≥35 g/L	43	43.8	0.871	12.9	0.409
≤35 g/L	52	48.7		38.2	
**Serum globulin level**					
≥35 g/L	32	38.5	0.495	19.3	0.037
<35 g/L	63	51.1		34.6	
**IPI score**					
L	6	66.7	0.005	50.0	0.009
LI	24	68.1		33.2	
HI	37	45.8		39.0	
H	33	17.2		8.8	
**PIT score**					
Group 1	9	64.8	0.000	33.3	0.045
Group 2	35	55.0		37.9	
Group 3	29	30.6		22.6	
Group 4	13	23.1		23.1	
Treatment*			0.816		0.763
Anthracycline-based regimens	64	45.1		26.4	
Gemcitabine and Platinum	30	47.5		36.5	
**Pathological finding**					
EBER					
Positive	42	51.7	0.529	26.7	0.376
Negative	15	58.7		50.0	
CXCL13					
Positive	46	37.9	0.348	26.4	0.919
Negative	18	55.9		30.5	
BCL-6					
Positive	59	41.7	0.730	42.9	0.778
Negative	7	57.1		22.2	
CD10					
Positive	54	48.7	0.427	26.2	0.937
Negative	23	37.3		32.8	
PD-1					
Positive	24	41.3	0.360	35.5	0.319
Negative	1	—		—

**Table 3:** Parameters influencing OS and PFS of AITL patients based on multivariate analysis.

Parameter	OS				PFS			
	P	Relative	95%CI	95%CI	P	Relative	95%CI	95%CI
		risk	low	high		risk	low	high
Rash/Pruritus	0.004	2.677	1.376	5.207	0.007	2.120	1.224	3.671
age >60	0.006	2.485	1.305	4.729	0.035	1.792	1.042	3.082
PS ≥2	0.006	2.889	1.358	6.147				
ENSs >1	0.029	1.946	1.069	3.544	0.006	2.038	1.226	3.388
β2-M ≥4.0	0.020	2.170	1.130	4.167				

Abbreviations: CI, Confidenceindex; PFS, progression-free survival; PS, performance status; OS, overall survival; β2-M, β2 microglobulin.

### The Prognostic Model

**Figure 2:**
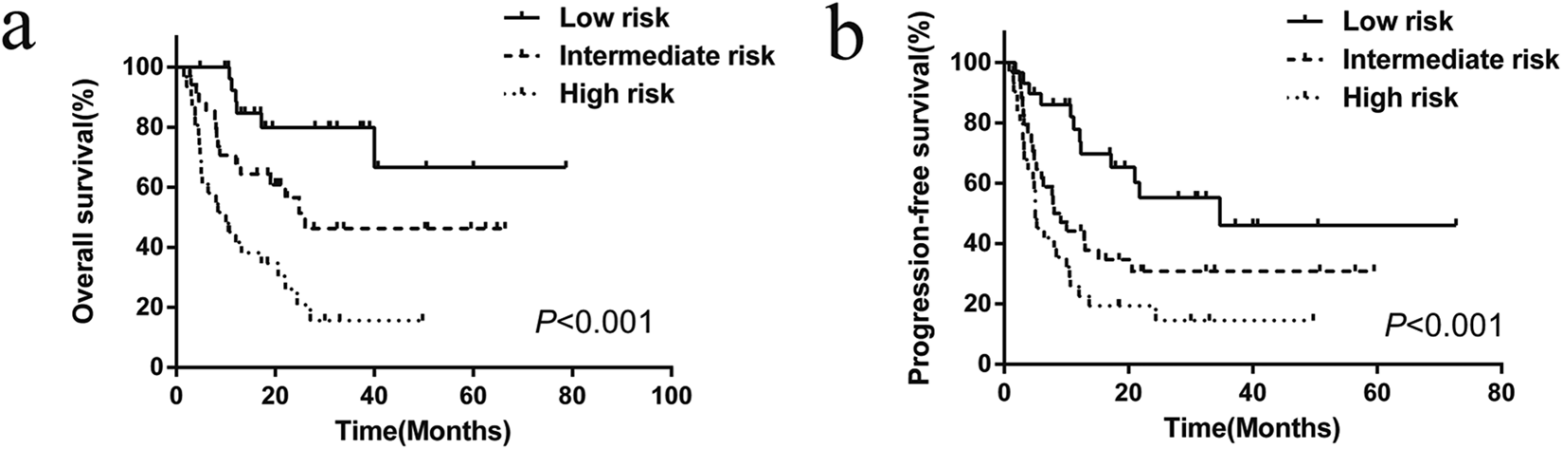
Overall survival (**a**) and progression-free survival (**b**) of 94 patients with angioimmunoblastic T-cell lymphoma according to our prognostic model. This prognostic model stratify patients into 3 groups: 0–1 risk factor (low-risk, n = 29), 2 risk factors (intermediate risk, n = 34), ≥3 risk factors (high risk, n = 31).

Based on the five indexes (β2-M ≥4.0 mg/L, rash/pruritus, age >60, PS ≥2 and ENSs >1), we tried to construct the prognostic model of AITL. The number of patients excluding those oral chemotherapy and untreated was 94 and they were classified into 3 risk stratifications with the following terms: low risk, 0–1 factor; intermediate risk, 2 factors; high risk, 3–5 factors. This novel prognostic model significantly put patients into different risk stratification (*P* < 0.001) (Fig. 2). Of the 94 patients, 29 (30.8%),34 (36.2%) and 31 (33.0%) patients were placed into the low-risk group, the intermediate-risk group and the high-risk group respectively, with the 3-year OS rates of 79.9%, 46.2% and 15.6% respectively. In terms of the 3-year PFS, the low-risk group, the intermediate-risk group and the high-risk group were 46.1%, 30.8% and 14.5% respectively (*P* < 0.001).

**Figure 3:**
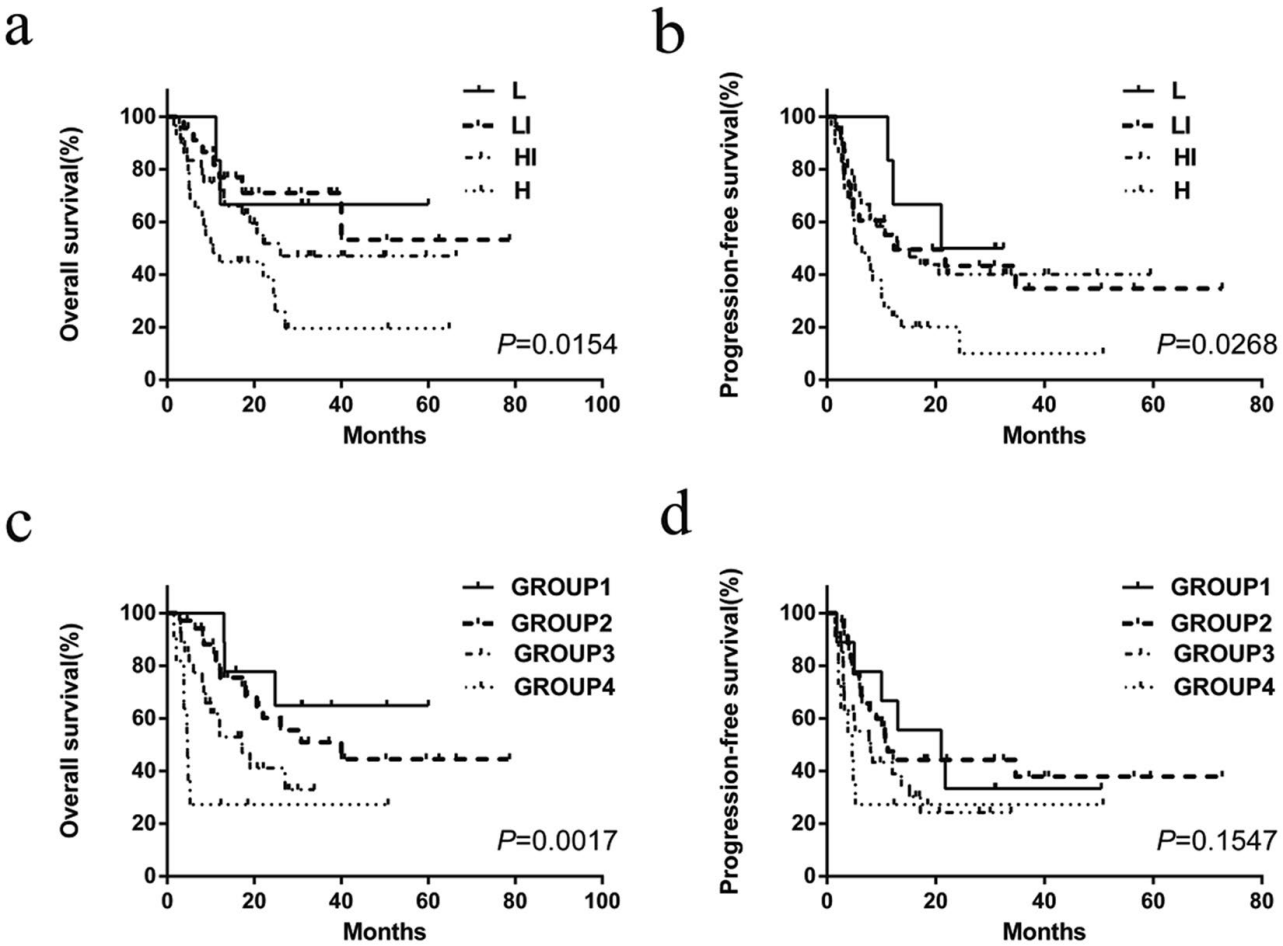
Overall survival and progression-free survival of 94 patients with angioimmunoblastic T-cell lymphoma according to the IPI (**a**,**b**) and PIT (**c**,**d**). L, low risk (6 patients); LI, low-intermediate risk (23 patients); HI, high-intermediate risk (36 patients); H, high risk (29 patients). PIT is categorized as follows: group 1 (0 risk factor, 9 patients), group 2 (1 risk factor, 35 patients), group 3 (2 risk factors, 27 patients), and group 4 (3–4 risk factors, 11 patients).

At the same time, the IPI and PIT were evaluated for OS and PFS using Kaplan–Meier curve analysis (Fig. 3). Compared with IPI and PIT, the receiver operating characteristic (ROC) curve area of our model was superior to those of IPI and PIT (our model ROC area, 0.747; 95%CI, 0.647–0.847; IPI ROC area, 0.661; 95% CI, 0.542–0.688; PIT ROC area, 0.622; 95% CI, 0.511–0.659). Furthermore, in terms of IPI, there was no statistical difference between low-intermediate risk group and high-intermediate risk group (*P* = 0.335).

## Discussion

Lymphoma is a kind of malignant tumor with strong heterogeneity and includes many subtypes with distinct prognosis. Though being a rare disease, AITL is the second most common subtype (18.5%) of peripheral T cell lymphoma or natural killer/T-cell lymphoma and has typical clinical and pathologic features different from other nodal peripheral T-cell lymphomas^3,5^. Studies showed that AITL originated from germinal center T-helper cells characteristically expressing CXCL13 and presented EBV infected cells. Use of the new immunostains for CXCL13 might help to improve diagnostic accuracy of AITL^18,19^. In present study, CXCL 13 (+) was in 74.0% and 71.0% cases contained a variable number of EBER-positive cells. However, it exerted no effect on survival whether or not presence of EBV-infected cells and expressing CXCL13, which was accordant with previous reports^3,4,19^.

The characteristics can be summarized as followers: More than half of the patients encountered high Ann Arbor stages (stage III/IV), extranodal involvement, B symptoms, anemia, lymphocytopenia, monocytoza, elevated LDH, elevated β2-M or hypoalbuminemia respectively, but all of them has no statistical significance to OS in univariate analysis except elevated β2-M. Rash/pruritus were observed in 29.9%.The two symptoms of rash/pruritus and rash/pruritus showed a significant correlation (*P* = 0.015) in present analysis. Spleen involvement (45.1%) is commonly seen than liver involvement (13.3%) or bone marrow involvement (16.0%), but only liver involvement, rather than the other two, is of statistical significance to OS in univariate analysis. Unlike other subtypes T cell lymphoma, AITL is inclined to suffer serious immunodeficiency. In recent 3 years, studies have certified that the frequent coexistence of somatic mutations in the Rho GTPase RhoA (RhoAG17V) and loss-of-function mutations in the 5-methylcytosine oxidase TET2 which may account for immunoinflammatory responses were associated with AITL^20,21^. On account of immunodeficiency, opportunistic infections increase and intense chemotherapy is difficult to conduct which also result in poor prognosis. In this study, the median survival time was 22 months and 3-year OS rate was 43.3%.

Although there are many known prognostic factors, such as the IPI and PIT, there are still no effective clinical indices that can be used for the prognostic stratification of AITL patients. IPI and PIT are controversial for the application to AITL. In this analysis, for IPI and PIT, it seems to be of statistical significance to prognostic stratification, but the factors such as the elevated LDH (OS:*P* = 0.166, PFS:*P* = 0.223), Ann Arbor stage(OS:*P* = 0.774, PFS:*P* = 0.887) and bone marrow involvement (OS:*P* = 0.063, PFS:*P* = 0.313) had no statistical significance to OS and PFS, which was in line with Federico M and Mourad N and other researchers^3,4,22,23^. Furthermore, there was no statistical difference between low-intermediate risk group and high-intermediate risk group of IPI (*P* = 0.335). So we aimed to explore prognostic factors and novel model to better evaluate prognosis. In this study, statistical analysis proved that elevated β2-M, rash/pruritus, PS ≥2, age ≥60 years and ENSs >1 were independent prognostic factors for AITL while β2-M and rash/pruritus were not used in IPI or PIT prognostic model. That we constructed novel prognosis model with β2-M (*P* = 0.020), rash/pruritus (*P* = 0.004), PS ≥2 (*P* = 0.006), age ≥60 years (*P* = 0.006) and ENSs >1(*P* = 0.029) perfectly stratified patients with AITL (Fig. 2a,b).

Several factors may support the established prognostic model. First of all, rash/pruritus is a relatively common clinical feature of AITL in the scale of lymphoma and was also regarded as one of the prognostic factors associated with shorter survival for AITL by Archimbaud^24,25^. In terms of β2-M, it has been reported to be adverse prognostic index in various subtypes of lymphoma^26–28^. Several decades years ago, Swan F, Jr.^29^ questioned Ann Arbor staging system and insisted that the serum levels of β2-M might provide a more precise system for defining risk groups in large-cell lymphomas. Khouri IF and Rodriguez J.^30,31^ also evaluated that the high β2-M at transplantation was an adverse prognostic factor in patients with diffuse mantle cell lymphoma and peripheral T-cell lymphoma after ASCT. B2-M being a potentially prognostic risk factor for AITL, the possible mechanisms are as follows: on the one hand, β2-M participates in immune recognition and loss of functional β2-M through genomic alterations or other yet uncharacterized mechanisms leads to lack of HLA-I expression and escape from CTL^11^. On the other hand, β2-M mirrors tumor burden in many tumors^29^. Above all, β2-M and rash/pruritus used in our model are reasonable. Besides, PS ≥2, age ≥60 years and ENSs >1 are the acknowledged unfavorable prognostic factors in lymphoma. Our model including general characteristic (age), distinct feature (rash/pruritus), general condition (PS), tumor involvement (ENSs) and laboratory examination (β2-M) is quite all-sided. Compared with IPI and PIT, the value of ROC curve area of our model was superior to those of IPI and PIT.

In this study, that the median survival time was 22 months indicates adverse prognosis. To date, the optimal therapy for patients with AITL remains unestablished. Although anthracycline-based regimens were commonly used as the first-line therapy, these regimens had disappointing results^8,23,32^. Consolidation ASCT at the first CR remains under debate^3,32–35^. A prospective randomized controlled trial about the efficacy and safety of GDPT with standard CHOP regimen for patients with newly diagnosed PTCL was carried out at our center between 2010 and 2016 and Li L demonstrated that GDPT arms were better than CHOP arms^36^. In this study, 64 patients were treated with Anthracycline-based regimens and 30 patients were treated with Gemcitabine and Platinum regimens. There was no difference between the two arms (*P* = 0.816). The negative result different from Li L may result from disequilibrium of the two treatment groups.

New treatment regimens have also been studied. It has been evaluated the efficacy and safety of Chidamide in relapsed or refractory PTCL and shown a favorable efficacy and an acceptable safety profile^37–39^. Because of the EBV-positive B cells in AITL, Rituximab has been reported effective and might provide a potential therapeutic target^40,41^. Yang J.^42^ discovered that some monoclonal antibodies (m-Abs) specific to human β2-M could induce apoptosis via recruiting MHC class I molecules to lipid rafts and activating Lyn and PLCg2. Such m-Abs may offer a possibly therapeutic approach to AITL on account of its immunodeficiency and aberrant β2-M protein expression. Shi C. also suggested that β2-M was a promising new therapeutic target for human cancers^43^. In recent 3 years, studies has demonstrated that recurrently hypermethylated genes involved in T-cell receptor signaling and T-cell differentiation likely contribute to lymphomagenesis in AITL and speculated that targeting of TCR-related events may hold promise for the treatment of TFH-derived lymphomas^44,45^.

In conclusion, aggressive AITL had poor prognosis and anthracycline-based regimens failed to improve survival. It is necessary to confirm whether immune modulation, antiangiogenic agents and therapeutic targets are effective to improve survival for AITL. Further studies will be needed to explore the pathogenesis and search new therapeutic targets. B2-M is an adverse prognostic factor in AITL independent of IPI or PIT and m-Abs specific to human β2-M may provide a potential therapeutic target. Our prognostic model significantly stratify patients with AITL, which makes it possible to accurately evaluate prognosis. Further studies are welcome to evaluate our model.
